# *N*-(4-Chloro­phen­yl)maleimide

**DOI:** 10.1107/S160053680803016X

**Published:** 2008-09-24

**Authors:** Rodolfo Moreno-Fuquen, Zulay Pardo-Botero, Javier Ellena

**Affiliations:** a Departamento de Química, Facultad de Ciencias, Universidad del Valle, Apartado 25360, Santiago de Cali, Colombia; b Instituto de Física de São Carlos, Universidade de São Paulo, USP, São Carlos, SP, Brazil

## Abstract

In the title compound, C_10_H_6_ClNO_2_, the dihedral angle between the benzene and maleimide rings is 47.54 (9)°. Mol­ecules form centrosymmetric dimers through C—H⋯O hydrogen bonds, resulting in rings of graph-set motif *R*_2_^2^(8) and chains in the [100] direction. Mol­ecules are also linked by C—H⋯Cl hydrogen bonds along [001]. In this same direction, mol­ecules are connected to other neighbouring mol­ecules by C—H⋯O hydrogen bonds, forming edge-fused *R*_4_^4^(24) rings.

## Related literature

For general background, see: Etter (1990 ▶); Howell & Zhang (2006 ▶); Miller *et al.* (2000 ▶, 2001 ▶); Moreno-Fuquen, Valencia, Abonia, Kennedy & Graham (2003 ▶); Nardelli (1995 ▶); Sarma & Desiraju (1986 ▶). 
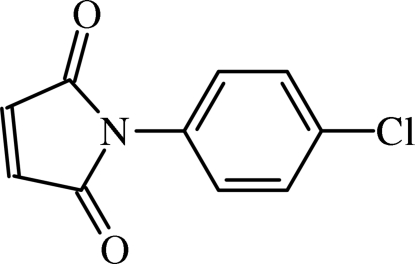


## Experimental

### 

#### Crystal data

C_10_H_6_ClNO_2_
*M* *_r_* = 207.61
Monoclinic, 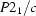

*a* = 10.6504 (7) Å
*b* = 3.8589 (2) Å
*c* = 22.0308 (14) Å
β = 100.741 (3)°
*V* = 889.57 (9) Å^3^
*Z* = 4
Mo *K*α radiation
μ = 0.40 mm^−1^
*T* = 150 K
0.18 × 0.04 × 0.03 mm

#### Data collection

Bruker–Nonius KappaCCD diffractometer
Absorption correction: multi-scan (*DENZO*; Otwinowski & Minor, 1997 ▶) *T*_min_ = 0.951, *T*_max_ = 0.982
11729 measured reflections
1646 independent reflections
1231 reflections with *I* > 2σ(*I*)
*R*_int_ = 0.089

#### Refinement

*R*[*F* ^2^ > 2σ(*F* ^2^)] = 0.042
*wR*(*F* ^2^) = 0.116
*S* = 1.07
1646 reflections
128 parameters
H-atom parameters constrained
Δρ_max_ = 0.21 e Å^−3^
Δρ_min_ = −0.31 e Å^−3^

### 

Data collection: *DENZO* (Otwinowski & Minor, 1997 ▶) and *COLLECT* (Nonius, 2000 ▶); cell refinement: *DENZO*; data reduction: *DENZO*; program(s) used to solve structure: *SHELXS97* (Sheldrick, 2008 ▶); program(s) used to refine structure: *SHELXL97* (Sheldrick, 2008 ▶); molecular graphics: *ORTEP-3 for Windows* (Farrugia, 1997 ▶); software used to prepare material for publication: *PARST95* (Nardelli, 1995 ▶).

## Supplementary Material

Crystal structure: contains datablocks I, global. DOI: 10.1107/S160053680803016X/fj2150sup1.cif

Structure factors: contains datablocks I. DOI: 10.1107/S160053680803016X/fj2150Isup2.hkl

Additional supplementary materials:  crystallographic information; 3D view; checkCIF report

## Figures and Tables

**Table 1:** Hydrogen-bond geometry (Å, °)

*D*—H⋯*A*	*D*—H	H⋯*A*	*D*⋯*A*	*D*—H⋯*A*
C8—H8⋯O1^i^	0.93	2.58	3.493 (3)	169
C2—H2⋯O1^ii^	0.93	2.77	3.659 (3)	161
C5—H5⋯O2^iii^	0.93	2.58	3.319 (3)	137
C9—H9⋯O2^iv^	0.93	2.64	3.326 (3)	131
C9—H9⋯Cl1^v^	0.93	2.89	3.551 (3)	129

Symmetry codes: (i) 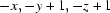; (ii) 
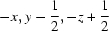
; (iii) 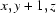; (iv) 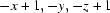; (v) 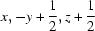.

**Fig. 1.**
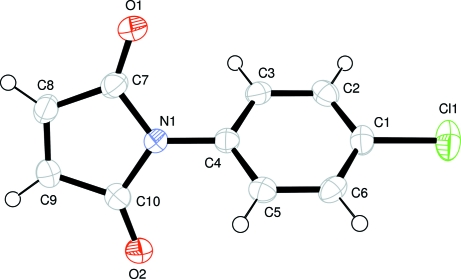
An ORTEP-3 (Farrugia, 1997) plot of the (I) compound, with the atomic labelling scheme. The shapes of the ellipsoids correspond to 50% probability contours of atomic displacement and, for the sake of clarity, H atoms are shown as spheres of arbitrary radius.

**Fig. 2.**
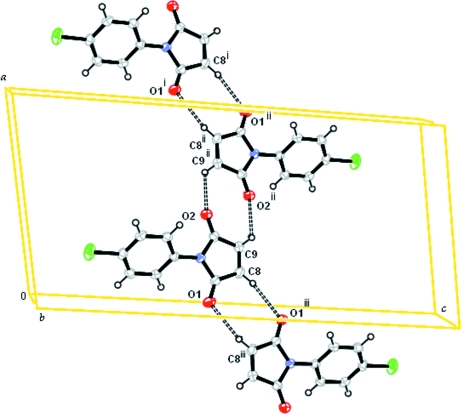
Part of the crystal structure of (I) showing the formation of centrosymmetric R22(8) dimmers rings and C(4) chains which are running parallel to the [100] direction. [Symmetry codes: (i) 1 + x,y + z; (ii)1 - x,1 - y,1 - z; (iii) -x,1 - y,1 - z].

**Fig. 3.**
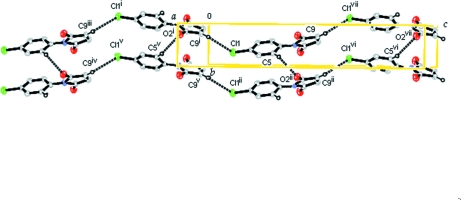
Part of the crystal structure of (I) showing the formation of C(9) chains and edge-fused R44(24) rings along [001]. [Symmetry codes: (i) x,1/2 - y,-1/2 + z; (ii) x,1 + y,z; (iii) x,y,-1 + z; (iv) x,1 + y,-1 + z; (v) x,3/2 - y,-1/2 + z; (vi) x,1/2 - y,1/2 + z; (vii) x,1/2 - y,1/2 + z].

**Fig. 4.**
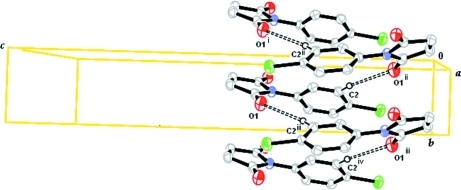
Part of the crystal structure of (I) showing the formation of C(7) chains along [010]. [Symmetry codes: (i) x,-1 + y,z; (ii) -x,-1/2 + y,1/2 + z; (iii) -x,1/2 + y,1/2 - z; (iv) x,1 + y,z].

**Fig. 5.**
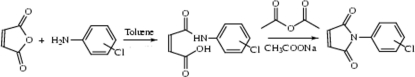
The formation of the title compound.

## supplementary crystallographic information

### Comment

It is known that cyclic unsaturated dicarbonyl compounds such as N-substituted maleimides can be used in free-radical-initiated polymerization processes upon exposure to light (Howell & Zhang, 2006). In order to study the possible application of *N*-(*p*-chlorophenylmaleimide) (I) in polymerization processes, and to explain its hydrogen bonding patterns, the synthesis and the study of the crystal structure are reported in this work. *N*-(*p*-nitrophenylmaleimide) (4NPMI) (Moreno-Fuquen *et al.*, 2003), *N*-(*o*-chlorophenylmaleimide) (2ClPMI) systems (Miller *et al.*, 2001) show a close analogy to the title compounds and are thus employed as a basic reference for comparison. Perspective view of (I), showing the atomic numbering scheme, can be seen in Fig.1. In the arylmaleimide systems the value of the dihedral angle between the benzene and imidic rings influences on the polimerization process, and the presence of different substituents in the benzene ring change the value of this angle (Miller *et al.*, 2000). The photochemical properties of arylmaleimide systems depend on the value of this angle. The dihedral angle between benzene and maleimide planes is 42.98 (5)° for 4NPMI, 66.10 (4) ° for 2ClPMI and 47.54 (9)° for (I). The chlorine atoms, which are pending on the aromatic nucleus, tend to steer the crystal structure to a state characterized by a short axis (Sarma & Desiraju, 1986). For (I), the *b* axis has a small value [3.8589 (2) Å] and a Cl···Cl nonbonded contact is observed at the same distance. The crystal structure of (I) is stabilized by weak intermolecular C—H···O and C—H···Cl hydrogen-bonds (Nardelli, 1995) (Table 1). The molecules of (I) are linked into a three-dimensional framework by a combination of C—H···O and C—H···Cl hydrogen bonds. The formation of the framework can be explained in terms of three-one substructures. In the first substructure, atom C8 in the molecule at (*x*,*y*,*z*) acts as a hydrogen-bond donor to maleimidic atom O1 in the molecule at (-*x*,1 - *y*,1 - *z*) and atom C9 in the molecule at (*x*,*y*,*z*) acts as a hydrogen-bond donor to maleimidic atom O2 in the molecule at (1 - *x*,1 - *y*,1 - *z*). Both interactions generate dimers containing centrosymmetric rings with graph motif *R*_2_^2^(8) (Etter, 1990) (Fig. 2, supp. material). These dimers are linked by C(5) chains which are running parallel to [100] direction. In the second substructure, atom C9 in the molecule at (*x*,1/2 - *y*,-1/2 + *z*) acts as a hydrogen-bond donor to atom Cl1 in the molecule at (*x*,*y*,*z*), similarly, atom C5 in the molecule at (*x*,*y*,*z*) acts as a hydrogen-bond donor to maleimidic atom O2 in the molecule at (*x*,1 + *y*,*z*) so generating a chain of edge-fused *R*_4_^4^(24) rings along [001] (Fig. 3, supp. material). The third one-dimensional substructure is built by C—H···O hydrogen bonds. Atom C2 in the molecule at (*x*,*y*,*z*) acts as hydrogen bond donor to maleimidic O1 in the molecule at (-*x*,-1/2 + *y*,1/2 - *z*) so generating a C(7) chains in the [010] direction (Fig.4, supp. material). The low value of the dihedral angle between benzene and maleimide planes, allows to conclude that (I) is not a good candidate to use in a photopolymerization process.

### Experimental

All reagents (purchased from Aldrich) and solvents were used as received. Column chromatography was performed using silica gel H60 to purify the intermediates and final products. Thin layer chromatography (TLC) was used to confirm the structure of the individual compounds.

### Refinement

The space group P 2_1_/c for (I) was uniquely assigned from the systematic absences. All H-atoms were located from difference maps and then treated as riding atoms [C—H= 0.93Å and *U*_iso_(H)= 1.2*U*_eq_(C)].

### Crystal data

**Table:**

C_10_H_6_ClNO_2_	*F*(000) = 424
*M**_r_* = 207.61	*D*_x_ = 1.550 Mg m^−^^3^
Monoclinic, *P*2_1_/*c*	Melting point: 384(1) K
Hall symbol: -P 2ybc	Mo *K*α radiation, λ = 0.71073 Å
*a* = 10.6504 (7) Å	Cell parameters from 11729 reflections
*b* = 3.8589 (2) Å	θ = 2.9–25.4°
*c* = 22.0308 (14) Å	µ = 0.40 mm^−^^1^
β = 100.741 (3)°	*T* = 150 K
*V* = 889.57 (9) Å^3^	Needle, pale-yellow
*Z* = 4	0.18 × 0.04 × 0.03 mm

### Data collection

**Table:**

Bruker–Nonius KappaCCD diffractometer	1646 independent reflections
Radiation source: fine-focus sealed tube	1231 reflections with *I* > 2σ(*I*)
graphite	*R*_int_ = 0.089
φ and ω scans	θ_max_ = 25.4°, θ_min_ = 3.0°
Absorption correction: multi-scan (DENZO; Otwinowski & Minor, 1997)	*h* = −12→12
*T*_min_ = 0.951, *T*_max_ = 0.982	*k* = −4→4
11729 measured reflections	*l* = −24→26

### Refinement

**Table:**

Refinement on *F*^2^	Primary atom site location: structure-invariant direct methods
Least-squares matrix: full	Secondary atom site location: difference Fourier map
*R*[*F*^2^ > 2σ(*F*^2^)] = 0.042	Hydrogen site location: inferred from neighbouring sites
*wR*(*F*^2^) = 0.116	H-atom parameters constrained
*S* = 1.07	*w* = 1/[σ^2^(*F*_o_^2^) + (0.0501*P*)^2^ + 0.491*P*] where *P* = (*F*_o_^2^ + 2*F*_c_^2^)/3
1646 reflections	(Δ/σ)_max_ < 0.001
128 parameters	Δρ_max_ = 0.21 e Å^−^^3^
0 restraints	Δρ_min_ = −0.31 e Å^−^^3^

### Special details

**Table:**

Geometry. All e.s.d.'s (except the e.s.d. in the dihedral angle between two l.s. planes) are estimated using the full covariance matrix. The cell e.s.d.'s are taken into account individually in the estimation of e.s.d.'s in distances, angles and torsion angles; correlations between e.s.d.'s in cell parameters are only used when they are defined by crystal symmetry. An approximate (isotropic) treatment of cell e.s.d.'s is used for estimating e.s.d.'s involving l.s. planes.

### Fractional atomic coordinates and isotropic or equivalent isotropic displacement parameters (Å^2^)

**Table:**

	*x*	*y*	*z*	*U*_iso_*/*U*_eq_	
Cl1	0.26337 (8)	0.6828 (2)	0.13972 (3)	0.0486 (3)	
O1	0.04581 (16)	0.6461 (5)	0.40894 (8)	0.0419 (5)	
O2	0.45773 (15)	0.2532 (5)	0.43624 (8)	0.0342 (5)	
N1	0.25175 (18)	0.4677 (5)	0.40446 (9)	0.0278 (5)	
C1	0.2590 (2)	0.6168 (6)	0.21732 (11)	0.0303 (6)	
C2	0.1564 (2)	0.4474 (7)	0.23409 (11)	0.0314 (6)	
H2	0.0899	0.3657	0.2040	0.038*	
C3	0.1537 (2)	0.4004 (6)	0.29608 (11)	0.0282 (6)	
H3	0.0852	0.2870	0.3081	0.034*	
C4	0.2542 (2)	0.5240 (6)	0.34041 (11)	0.0269 (6)	
C5	0.3567 (2)	0.6933 (6)	0.32329 (12)	0.0283 (6)	
H5	0.4237	0.7748	0.3532	0.034*	
C6	0.3585 (2)	0.7399 (6)	0.26132 (12)	0.0303 (6)	
H6	0.4266	0.8541	0.2492	0.036*	
C7	0.1462 (2)	0.5170 (7)	0.43329 (12)	0.0323 (6)	
C8	0.1852 (2)	0.3901 (7)	0.49762 (11)	0.0340 (6)	
H8	0.1336	0.3862	0.5273	0.041*	
C9	0.3057 (2)	0.2833 (7)	0.50601 (12)	0.0317 (6)	
H9	0.3524	0.1958	0.5427	0.038*	
C10	0.3538 (2)	0.3260 (6)	0.44722 (11)	0.0291 (6)	

### Atomic displacement parameters (Å^2^)

**Table:**

	*U*^11^	*U*^22^	*U*^33^	*U*^12^	*U*^13^	*U*^23^
Cl1	0.0730 (6)	0.0459 (5)	0.0305 (4)	0.0071 (4)	0.0186 (4)	0.0053 (3)
O1	0.0297 (10)	0.0600 (13)	0.0376 (11)	0.0126 (9)	0.0101 (8)	0.0056 (9)
O2	0.0285 (10)	0.0420 (11)	0.0320 (10)	0.0049 (8)	0.0055 (8)	−0.0004 (8)
N1	0.0251 (11)	0.0339 (12)	0.0249 (11)	0.0031 (9)	0.0061 (9)	0.0013 (9)
C1	0.0401 (15)	0.0260 (14)	0.0262 (14)	0.0073 (11)	0.0102 (11)	0.0018 (11)
C2	0.0325 (14)	0.0296 (14)	0.0304 (15)	0.0052 (11)	0.0009 (11)	−0.0018 (11)
C3	0.0242 (13)	0.0275 (14)	0.0337 (14)	0.0016 (10)	0.0074 (11)	0.0025 (11)
C4	0.0274 (13)	0.0262 (13)	0.0277 (13)	0.0047 (10)	0.0064 (10)	0.0008 (10)
C5	0.0278 (13)	0.0252 (13)	0.0316 (14)	0.0035 (10)	0.0045 (10)	−0.0025 (11)
C6	0.0301 (13)	0.0260 (14)	0.0382 (16)	0.0034 (11)	0.0151 (12)	0.0020 (11)
C7	0.0286 (14)	0.0368 (15)	0.0329 (15)	0.0005 (12)	0.0097 (11)	−0.0052 (12)
C8	0.0361 (15)	0.0392 (16)	0.0288 (14)	−0.0014 (12)	0.0121 (11)	−0.0005 (12)
C9	0.0346 (15)	0.0338 (14)	0.0260 (14)	−0.0009 (12)	0.0036 (11)	−0.0013 (11)
C10	0.0311 (14)	0.0261 (13)	0.0292 (14)	0.0017 (11)	0.0036 (11)	−0.0025 (11)

### Geometric parameters (Å, °)

**Table:**

Cl1—C1	1.738 (2)	C3—H3	0.9300
O1—C7	1.210 (3)	C4—C5	1.384 (3)
O2—C10	1.209 (3)	C5—C6	1.380 (4)
N1—C7	1.404 (3)	C5—H5	0.9300
N1—C10	1.410 (3)	C6—H6	0.9300
N1—C4	1.433 (3)	C7—C8	1.484 (4)
C1—C6	1.380 (4)	C8—C9	1.327 (4)
C1—C2	1.381 (4)	C8—H8	0.9300
C2—C3	1.383 (3)	C9—C10	1.489 (4)
C2—H2	0.9300	C9—H9	0.9300
C3—C4	1.392 (3)		
			
C7—N1—C10	109.5 (2)	C4—C5—H5	120.4
C7—N1—C4	126.0 (2)	C1—C6—C5	120.0 (2)
C10—N1—C4	124.3 (2)	C1—C6—H6	120.0
C6—C1—C2	121.1 (2)	C5—C6—H6	120.0
C6—C1—Cl1	118.86 (19)	O1—C7—N1	124.8 (2)
C2—C1—Cl1	120.01 (19)	O1—C7—C8	128.8 (2)
C1—C2—C3	119.3 (2)	N1—C7—C8	106.4 (2)
C1—C2—H2	120.4	C9—C8—C7	109.1 (2)
C3—C2—H2	120.4	C9—C8—H8	125.5
C2—C3—C4	119.5 (2)	C7—C8—H8	125.5
C2—C3—H3	120.2	C8—C9—C10	109.0 (2)
C4—C3—H3	120.2	C8—C9—H9	125.5
C5—C4—C3	120.9 (2)	C10—C9—H9	125.5
C5—C4—N1	120.0 (2)	O2—C10—N1	125.3 (2)
C3—C4—N1	119.1 (2)	O2—C10—C9	128.7 (2)
C6—C5—C4	119.2 (2)	N1—C10—C9	106.0 (2)
C6—C5—H5	120.4		
			
C6—C1—C2—C3	0.0 (4)	C10—N1—C7—O1	177.3 (3)
Cl1—C1—C2—C3	−179.27 (18)	C4—N1—C7—O1	−7.5 (4)
C1—C2—C3—C4	−0.1 (4)	C10—N1—C7—C8	−1.2 (3)
C2—C3—C4—C5	0.0 (4)	C4—N1—C7—C8	174.0 (2)
C2—C3—C4—N1	−178.7 (2)	O1—C7—C8—C9	−177.0 (3)
C7—N1—C4—C5	136.1 (3)	N1—C7—C8—C9	1.5 (3)
C10—N1—C4—C5	−49.4 (3)	C7—C8—C9—C10	−1.1 (3)
C7—N1—C4—C3	−45.1 (3)	C7—N1—C10—O2	179.8 (2)
C10—N1—C4—C3	129.4 (3)	C4—N1—C10—O2	4.4 (4)
C3—C4—C5—C6	0.1 (4)	C7—N1—C10—C9	0.6 (3)
N1—C4—C5—C6	178.9 (2)	C4—N1—C10—C9	−174.7 (2)
C2—C1—C6—C5	0.2 (4)	C8—C9—C10—O2	−178.8 (3)
Cl1—C1—C6—C5	179.46 (18)	C8—C9—C10—N1	0.3 (3)
C4—C5—C6—C1	−0.3 (4)		

### Hydrogen-bond geometry (Å, °)

**Table:**

*D*—H···*A*	*D*—H	H···*A*	*D*···*A*	*D*—H···*A*
C8—H8···O1^i^	0.93	2.58	3.493 (3)	169
C2—H2···O1^ii^	0.93	2.77	3.659 (3)	161
C5—H5···O2^iii^	0.93	2.58	3.319 (3)	137
C9—H9···O2^iv^	0.93	2.64	3.326 (3)	131
C9—H9···Cl1^v^	0.93	2.89	3.551 (3)	129

Symmetry codes: (i) −*x*, −*y*+1, −*z*+1; (ii) −*x*, *y*−1/2, −*z*+1/2; (iii) *x*, *y*+1, *z*; (iv) −*x*+1, −*y*, −*z*+1; (v) *x*, −*y*+1/2, *z*+1/2.
